# Investigation of fungal biomolecules after Low Earth Orbit exposure: a testbed for the next Moon missions

**DOI:** 10.1111/1462-2920.15995

**Published:** 2022-04-22

**Authors:** Alessia Cassaro, Claudia Pacelli, Mickael Baqué, Barbara Cavalazzi, Giorgio Gasparotto, Raffaele Saladino, Lorenzo Botta, Ute Böttger, Elke Rabbow, Jean‐Pierre de Vera, Silvano Onofri

**Affiliations:** ^1^ Department of Ecological and Biological Sciences University of Tuscia, Largo dell'Università snc Viterbo 01100 Italy; ^2^ Human Spaceflight and Scientific Research Unit Italian Space Agency, via del Politecnico Rome 00133 Italy; ^3^ German Aerospace Center (DLR), Institute of Planetary Research, Planetary Laboratories Department, Rutherfordstraße 2 Berlin Germany; ^4^ Department of Biological, Geological and Environmental Sciences University of Bologna, Via Zamboni 67 Bologna 40126 Italy; ^5^ Department of Geology University of Johannesburg, Auckland Park Johannesburg 2006 South Africa; ^6^ Le Studium Loire Valley Institute for Advanced Studies, Rue Dupanloup 1 Orléans France; ^7^ German Aerospace Center (DLR), Institute of Optical Sensor Systems, Rutherfordstraße 2 Berlin Germany; ^8^ German Aerospace Center (DLR), Institute of Aerospace Medicine, Radiation Biology, Linder Höhe Cologne 51147 Germany; ^9^ Space Operations and Astronaut Training, MUSC, German Aerospace Center (DLR), Linder Höhe Cologne 51147 Germany

## Abstract

The Moon is characterized by extremely harsh conditions due to ultraviolet irradiation, wide temperature extremes, vacuum resulting from the absence of an atmosphere and high ionizing radiation. Therefore, its surface may provide a unique platform to investigate the effects of such conditions. For lunar exploration with the Lunar Gateway platform, exposure experiments in Low Earth Orbit are useful testbeds to prepare for lunar space experiments and to understand how and if potential biomarkers are influenced by extra‐terrestrial conditions. During the BIOMEX (BIOlogy and Mars EXperiment) project, dried colonies of the fungus *Cryomyces antarcticus* grown on Lunar Regolith Analogue (LRA) were exposed to space conditions for 16 months aboard the EXPOSE‐R2 payload outside the International Space Station. In this study, we investigated the stability/degradation of fungal biomarkers in LRA after exposure to (i) simulated space and (ii) real space conditions, using Raman spectroscopy, gas chromatography–mass spectrometry and DNA amplification. The results demonstrated that fungal biomarkers were detectable after 16 months of real space exposure. This work will contribute to the interpretation of data from future biological experiments in the Cislunar orbit with the Lunar Gateway platform and/or on the lunar surface, in preparation for the next step of human exploration.

## Introduction

Fifty years after the first human set foot on the Moon, lunar exploration is back in the spotlight of space agencies for crewed and *in situ* missions. Exploration of the moon began before the 1960s, with the Soviet Union Luna program. From 1969 to 1976, the NASA Apollo program carried out a series of crewed missions, bringing humans to the Moon and returning them back to the Earth. Fourteen years after the end of the Apollo program, Japan sent the Hiten and SELENE spacecraft to the Moon, in 1990 and 2007 respectively, with the aim of obtaining information about its origin and evolution. In the same year, the Chinese lunar exploration program began with the launch of the Chang'e 1 lunar orbiter. In 2003, the European Space Agency launched the lunar orbital probe SMART‐1 to acquire spectroscopic images of the lunar surface. Owing to the many missions around and on it, currently, the Moon can be considered the only body beyond Earth that has been partially explored and sampled, despite its extreme conditions (Vaniman *et al*., 2003).

The lunar atmosphere is described as a hard vacuum, with a gas concentration in the range of 10^5^–10^4^ molecules cm^−3^ (Vaniman *et al*., 2003). The lunar surface is extremely harsh and is characterized by temperature fluctuations between −171°C and 140°C (Langseth *et al*., 1972), pressure values that may reach 10^−10^ Pa (Schuerger, 2004), high doses of ultraviolet (UV) irradiation (26.8 W m^−2^ UVC/UVB) and high levels of ionizing radiation (Schuerger *et al*., 2019). Despite these prohibitive conditions, the Moon is the next step forward in human exploration, a place to develop the ability for advanced space exploration. The lunar surface serves as a training ground for testing instrumentation for future missions to Mars and other destinations. For instance, the Moon offers an ideal platform for studies related to the origin, evolution and distribution of life. The next step back to the Moon will include the NASA Artemis Program, which aims to land a human on the Moon by 2024 (https://www.nasa.gov/specials/artemis/), and the development of the Lunar Gateway platform, an orbital human outpost that will explore the lunar surface and its resources (https://www.nasa.gov/gateway).

In addition, the idea of lunar exploration to study signs of life in an extreme environment and to deepen knowledge about the development of life on Earth arises from some intrinsic features. First, the lack of recent geological activity and active erosional processes could have locally maintained the lunar surface with characteristics similar to those of 3 and 4.5 billion years ago (Jaumann *et al*., 2012), allowing the preservation of records of the early geological evolution of the Earth (Crawford *et al*., 2012). The lunar surface can also preserve evidence, such as meteorites, solar wind flux and composition, and galactic cosmic ray flux, allowing an understanding of the past habitability of our own planet (Crawford, 2006; Cockell, 2010). Furthermore, according to the Lithopanspermia theory, the phenomena of exchange of colonized rock fragments between the Earth and Moon could have been aided by the lunar atmosphere (which first appeared about 3.5 Ga ago and has a lifetime of about 70 million years; Needham and Kring, 2017), reducing the impact of such rocks on the lunar environment.

In the astrobiological scenario, the Moon also provides a unique experimental platform to investigate the effects of ionizing and cosmic radiations, solar energetic particles, and minimal magnetic shielding on biological components. The future launch of the Lunar Gateway spaceship will allow the scientific community to perform several experiments in deep space and the Cislunar orbit. Since the first astrobiological experiments in the Cislunar orbit and on the lunar surface will require a few years, experiments in Low Earth Orbit (LEO) are baseline tests to improve our capability to perform experiments in a completely different and more hostile environment.

In this context, the melanized fungus *Cryomyces antarcticus* was selected as a test organism for astrobiological studies owing to its extraordinary resistance to stress (Onofri *et al*., 2012, 2015, 2019; Pacelli *et al*., 2017, 2019; Aureli *et al*., 2020; Pacelli *et al*., 2020a; Pacelli *et al*., 2020b; Pacelli *et al*., 2021; Cassaro *et al*., 2021a). After being grown on Lunar Regolith Analogue (LRA), this fungus was employed in the BIOMEX (BIOlogy and Mars Experiment) project, which aimed to investigate the detectability of biomarkers and the viability of different microorganisms after real space exposure (de Vera *et al*., 2012). The ongoing and future life‐detection missions will be finalized to search for signs of life [named biomarkers, which may be defined as ‘an object, substance and/or pattern whose origin specifically requires a biological agent’ (Des Marais *et al*., 2008)] beyond Earth. Starting from this definition, such substances can be defined as materials or combinations, including elemental abundances, molecules, allotropes and enantiomers (Chan *et al*., 2019). Terrestrial life is still the only known example of life and molecules from cells – building blocks of terrestrial life – are the only reference we can account for. For example, because nucleic acids from all known terrestrial life are used as genetic material, its detection would unambiguously prove the presence of life and could indicate an organism with common ancestries to those on Earth (Mojarro *et al*., 2017; Pontefract *et al*., 2017). In addition, melanin pigments are adopted from many organisms in all the kingdoms of life, and in the last decade, their importance as biomarkers has increased (Pacelli *et al*., 2021). The exposure of pigmented microorganisms to simulated space or Martian conditions suggested the higher tolerance of these species and their biomolecules compared to their non‐melanized counterparts. Melanin pigments play a strong role in protecting against ionizing and non‐ionizing radiation (Cordero and Casadevall, 2017), and hence, may be exploited as protective organic materials during human space missions. Despite this, the high instability of biological molecules in an environment not protected from a magnetic field (radiation is one of the main limiting factors for life detection because of the induction of direct or indirect effects on biomolecules) has been widely reported. Therefore, preliminary tests to evaluate biomolecule stability are needed.

In this context, dried colonies of *C*. *antarcticus* were exposed to (i) simulated space conditions, during the Science Verification Tests (SVTs), a series of pre‐flight ground experiments (referred to as ‘SVT’ samples), and (ii) real space in LEO (referred to as ‘SPACE’ samples), within the EXPOSE‐R2 facility for 16 months aboard the International Space Station (ISS). With the aim of understanding the stability of fungal biomarkers after exposure to real space conditions, we investigated the role of lunar analogue minerals in protecting/degrading fungal biomarkers, in addition to studying the effects of Mars analogue mineral mixtures. These studies allowed us to compare the effects of simulated space and real space conditions on biomolecules using Raman spectroscopy and gas chromatography–mass spectrometry (GC–MS), with two instruments aboard the scientific payloads of the Rosalind Franklin and Perseverance rovers on Mars, as well as through quantitative DNA amplification analyses. A specific focus on the LRA study was necessary to understand if regolith could interfere with the extraction and detection of biomarkers or act as a protective layer against the stressful conditions of the surface. The LRA was further characterized to better define the main mineral phases and to compare it with the Apollo 17 samples.

## Results

### Mineralogical composition of LRA: XRD, XRF and SEM‐EDS results

X‐ray powder diffraction (XRD) of the LRA indicated the following dominant phases: 97.5% Na‐plagioclase (Na,Ca)AlSi_3_O_8_. The concentrations (wt.%) of oxides were determined through X‐ray fluorescence (XRF) analysis (Table S1), and compared with the composition of lunar samples collected during the Apollo 17 mission. Similar oxides were found in the Apollo 17 samples (Table S1). Scanning electron microscopy coupled with dispersive X‐ray spectrometry (SEM‐EDS) characterization allowed elemental analyses and identification of the following dominant minerals and volcanic glass: olivine, plagioclase, diopside, ilmenite, iron oxides, hypersthene and apatite (Fig. S1).

### Detection of possible alterations in melanin pigments by confocal Raman spectroscopy and heat map analyses

**Fig. 1:**
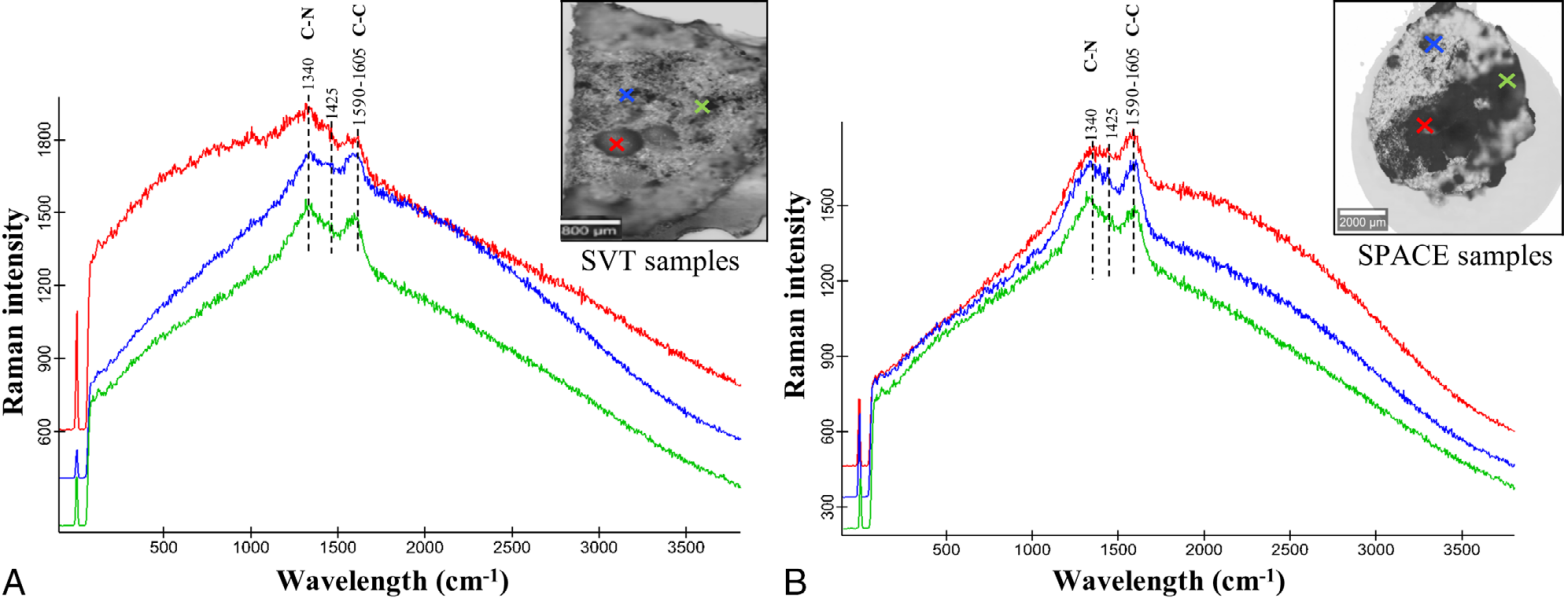
Raman spectra of melanin pigments (main peaks at 1340, 1425 and 1590–1605 cm^−1^) from *C*. *antarcticus* colonies grown on LRA and exposed to (A) simulated space conditions (SVT samples), and (B) real space conditions (SPACE samples). Red spectrum = samples exposed to radiation with 0.1% neutral density filters (Top); blue spectrum = dark control samples (Bottom); green spectrum = samples kept in the laboratory in the dark at room temperature (Control).

Colonies of *C*. *antarcticus* grown on LRA were analysed using confocal Raman spectroscopy after exposure to simulated space (SVT samples) and real space conditions (SPACE samples). The results revealed a specific fingerprint of fungal colonies with two main peaks: an intense and broad peak at 1590–1605 cm^−1^ and a second peak at a low wavelength near 1340 cm^−1^ (Figs. 1A and B). According to Culka *et al*. (2017), these spectra are associated with the presence of melanin pigments. The presence of these peaks is probably due to the aromatic C‐N bonds, represented by the first peak (Samokhvalov *et al*., 2004), and the stretching of the C‐C bonds within the rings of the aromatic melanin monomers, represented by the second peak (Galván *et al*., 2013). The peculiar feature of the *C*. *antarcticus* melanin spectrum is an additional peak at approximately 1425 cm^−1^, which indicates melanin pigments from burnt organic matter and/or amorphous carbon, as previously reported by Pacelli *et al*. (2021). Figure S2A and B show the signal coverage for the image scan analyses of the SVT and SPACE samples respectively, calculated by the application of a signal‐to‐noise ratio (SNR) mask greater than 5. The results were normalized to the corresponding SNR of the non‐irradiated samples (control samples). Despite the treatments, no significant melanin changes were observed in any of the collected spectra.

In this work, we used heat map (HM) graphics to represent differences among the main peak positions in the melanin Raman spectra (approximately 1600 cm^−1^), where the columns correspond to the Top (exposed in the upper layer of the EXPOSE‐R2 payload; additional information is reported in the Experimental procedures section), Bottom (kept in the dark during the exposure within the second layer of the EXPOSE‐R2 payload, experiencing all parameters except UV radiation. Additional information is reported in the Experimental procedures section), and Control (laboratory samples; not exposed) exposure conditions of the SVT and SPACE samples (Fig. S3), and the rows of the grid correspond to each wavelength value. Different colours (in red‐green scale) cover the entire range of spectral intensities, and the difference in colour intensity of each row is due to the different values across the spectra. Our results revealed similar peak positions (1602–1604 cm^−1^) for the SVT and SPACE samples (Fig. S3A and B respectively) for both Bottom and Top exposure, in comparison with the control samples. The peak position of the Bottom exposure conditions of the SVT samples is at a slightly shorter wavelength than that of the Top and Control conditions (Fig. S3A), and the same shift was found for the Bottom exposure of the SPACE samples (Fig. S3B).

### Detection of melanin pigments by spectrophotometric analyses

**Fig. 2:**
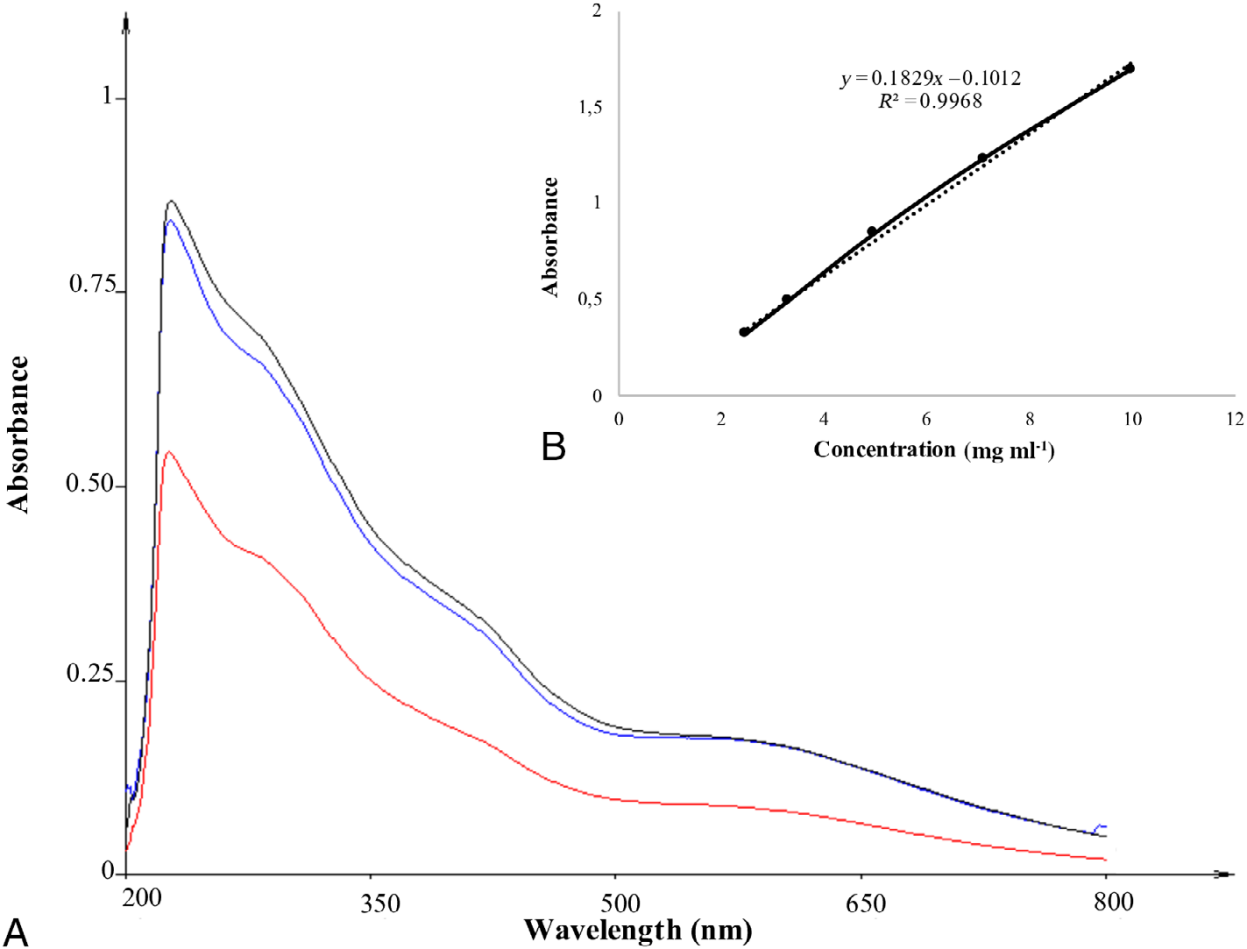
A. UV–Vis spectra of melanin pigment extracted from *C*. *antarcticus* colonies grown on LRA exposed to simulated space conditions (SVT samples). Red spectrum = samples exposed to radiation with 0.1% neutral density filters (Top); blue spectrum = dark control samples (Bottom); black spectrum = samples kept in the laboratory in the dark at room temperature (Control). *X*‐axis, UV–Vis wavelengths (in nm); *Y*‐axis, absorbance values (in arbitrary units). B. Correlation between synthetic DHN melanin at five concentrations and absorbance at 650 nm, used as standard curve for quantification, according to Raman and Ramasamy (2017).

Since spectrophotometric analysis is a destructive technique and the number of samples exposed to space conditions was limited, melanin was extracted only from the SVT samples. Spectrophotometric analyses were performed at UV–Vis wavelengths in the range of 200–800 nm. Among all biological pigments, only melanin absorbs all visible wavelengths, owing to its dark colour (Bell and Wheeler, 1986). Overall, the most significant feature of fungal melanin is its intense absorption in the UV region, with a maximum absorbance value at ~230 nm, which progressively decreases as the wavelength increases; this is characteristic of melanin pigments (Fig. 2A). In addition, the melanin absorption spectra revealed a decreasing trend in the range of 270–280 nm, which may be due to the aromatic amino acids in melanin molecules (Sun *et al*., 2016).

SVT exposure did not alter the spectral properties of the extracted pigments, demonstrating high stability and confirming the detectability of melanin pigments. The concentration of extracted melanin was estimated by comparing the absorbance value at 650 nm of each sample with the *A*_650_ standard curve of synthetic DHN (1,8‐DiHydroxyNaphthalene) melanin (Fig. 2B), according to the protocol of Pacelli *et al*. (2020a). The concentrations are listed in Table S2.

### Low‐molecular‐weight organic compound detection by GC–MS

One of the instruments chosen for *in situ* planetary exploration is a gas chromatograph coupled with a mass spectrometer, which separates the volatile compounds produced from the heating or chemical treatment of the samples and provides a spectral signature for each compound. GC–MS has been widely used for space exploration, and is planned to be used for investigating lunar soil samples in the framework of Roscosmo's Luna‐Glob program (Hofer *et al*., 2015). We used this approach on fungal colonies grown on LRA after exposure to SVT (Top and Control exposure for SVT samples) and SPACE conditions (Top, Bottom, and Control exposure for SPACE samples). GC–MS analyses were performed only to compare the effects of the Top and Control conditions on the SVT and SPACE samples.

The analysis revealed the presence of several compounds: azelaic acid (1,7‐epta‐didecanoic acid), gentisic acid (2,5‐dihydroxybenzoic acid), lactic acid (2‐hydroxypropanoic acid), palmitic acid (hexadecanoic acid), glucose, fructose, glucitol, glycerol (1,2,3‐trihydroxy propane) and ethylene glycol (1,2‐dihydroxy ethane). The identified compounds are shown in Table 1 and the *m*/*z* fragmentation spectra of each identified compound are presented in Table S3.

**Table 1:** Abundance of main compounds detected with GC–MS analysis after SVT and SPACE exposure.

	Samples exposed to simulated space conditions (SVT)		Samples exposed to real space conditions (SPACE)	
Compounds^a^	Top	Control	Top	Control
Azelaic acid	0.06	0.90	0.08	nd
Gentisic acid	nd	nd	nd	0.26
Lactic acid	0.17	nd	0.22	0.24
Palmitic acid	nd	nd	0.52	1.15
Glucose	1.27	0.68	nd	nd
Fructose	3.31	2.50	0.21	nd
Glucitol	1.21	1.03	2.68	1.09
Glycerol	0.32	nd	nd	nd
Ethylene glycol	0.49	nd	nd	0.21

^a^ Analyses were performed after silylation with *N*,*N*‐bis‐trimethylsilyl trifluoroacetamide in pyridine (620 μl) at 60°C for 4 h in the presence of betulinic acid [3β‐3‐hydroxy‐lup‐20(29)‐en‐28‐oic acid] as the internal standard (0.2 mg). All quantities are expressed in μg. GC–MS analyses of lunar samples. Top samples exposed in the top of the payload in comparison with Control samples, not exposed to treatments. nd: not determined.

The results of the GC–MS analyses showed the presence of azelaic acid after the Top and Control exposure of SVT samples, and only after the Top exposure of SPACE samples. In addition, the amount of azelaic acid in the SVT samples decreased after exposure (Table 1). Gentisic acid was detected only in the SPACE samples after Control exposure. It was not detected in the SVT samples (Table 1). Palmitic acid was found only in the SPACE samples, both for the Control and Top exposure (Table 1). Surprisingly, the amount of palmitic acid decreased as the stress increased (Table 1). Glucose and fructose were present in the SVT samples, whereas only fructose was detected in the SPACE samples. According to Pacelli *et al*. (2021), low‐molecular‐weight carboxylic acids, such as pyruvic acid and lactic acid, are most likely produced by the radiolysis of glucose and fructose occurring in the growth medium. Glucitol was detected in both sets of samples (SVT and SPACE); a larger amount was detected after an increase in stress (Top exposure). A small amount of glycerol was found only in SVT samples upon Top exposure (Table 1).

### Investigation of nucleic acids by qPCR assay and relative amount of DNA lesions

The most commonly used method to detect DNA on Earth is based on the PCR assay. This procedure, although not planned for the imminent exploration mission, has been proposed for *in situ* life detection (Isenbarger *et al*., 2008), based on the theories that life in the Universe can share common features and that nucleic acids are among the few molecules which may provide unambiguous evidence of life (Mojarro *et al*., 2019). Owing to its sensitivity, specificity and accuracy (Raeymaekers, 2000), quantitative PCR was used to investigate any possible damage to DNA extracted from the SVT and SPACE samples.

**Fig. 3:**
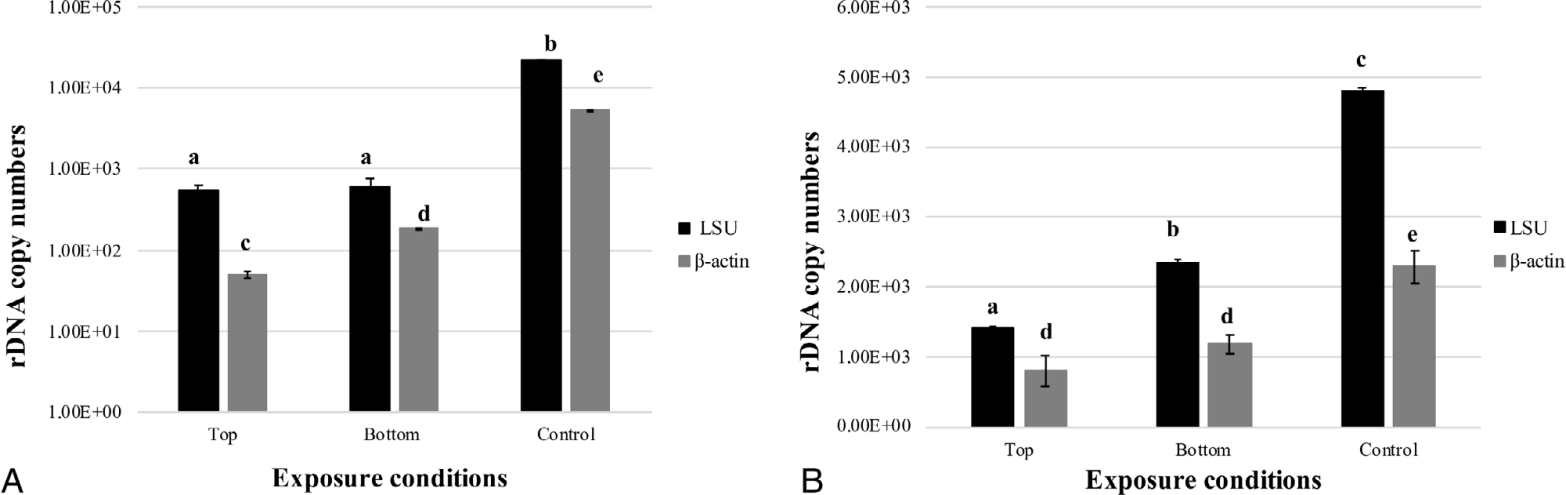
Comparison using quantitative PCR of a 939 bp target gene (LSU, black bars) and a 330 bp target gene (β‐actin, grey bars) of *C*. *antarcticus* DNA after exposure to (A) simulated space conditions (SVT samples) and (B) real space conditions (SPACE samples). On the *Y*‐axis, the number of amplified copies is shown in a logarithmic scale; the *X*‐axis indicates the treatments reported in Fig. 2. The same letters above the bars indicate that the values are not statistically different according to the *t‐*test (*p* ≤ 0.05).

Overall, the experiment assumes that damaged DNA cannot be amplified. Amplification was performed on a 939 bp gene spanning the ribosomal large subunit (LSU). Since ribosomal genes occur in multiple copies in the genome, we decided to amplify a region of 330 bp of the housekeeping gene (that of β‐actin), which is present in a single copy in the genome, to compare the level of detection based on the different gene lengths and number of copies in the genome. Figure 3A and B show the amplification of the 939 bp LSU gene and 330 bp β‐actin gene respectively. The results showed a high amount of amplified DNA under all the experimental conditions, and despite the amplification, 5260 and 1650 DNA copies on average for DNA extracted from SVT and SPACE samples respectively; not less than 10^2^ copies were amplified (Fig. 3). The test highlighted a common trend for all the samples, that is, a lower amount of amplified DNA for samples exposed to UV radiation and vacuum (Top exposure), and a higher copy number for samples exposed to vacuum but no radiation (Bottom exposure).

Overall, DNA was successfully amplified for all tested conditions, and the number of amplified DNA copies was never below the amplification limit, even when using the β‐actin gene, which is present as a single copy in the fungal genome.

The relative amplification rate (comparison between amplification of exposed samples and control) was calculated using the means from the amplification values (Table S4, column 2). The relative amount of amplified DNA (Table S4, column 4) decreased with an increase in stress (Top and Bottom exposure). The maximum amounts of lesions, calculated by Poisson distribution (~0.9 and 0.33 kb fragments, Fig. S4; Table S4, column 6), were reached after Top exposure of the SPACE and SVT samples respectively. Overall, the Poisson distribution highlighted more damage as stress increased (Top and Bottom exposure); more DNA damage was detected during β‐actin gene amplification of DNA extracted from SVT samples, and similar DNA damage was detected during LSU and β‐actin gene amplification of DNA extracted from SPACE samples (Table S4, column 6).

## Discussion

In the frame of planetary exploration, the Moon is of great astrobiological interest because, given the limited volcanic activity, its surface could preserve a geological record of conditions in the Earth‐Moon system during the first billion years of the history of our solar system. Although the Moon has never been considered to have supported any form of life on its own, it may provide insights for long‐term space missions and on the relationship between the extreme conditions of planetary surfaces and microbial survival. The growing interest in lunar exploration would permit further study of Earth life forms in an environment different from that experienced outside the ISS, as the Moon is outside the protection of Earth's geomagnetic field. Moreover, it is important to study the behaviour of terrestrial life forms or biological molecules in a different scenario because on the Moon, hypothetical life forms could be subjected to the dangerous interaction of the LRA with solar winds and galactic cosmic rays (Heinicke and Foing, 2021).

Future experiments on the Moon will provide new insights into the (i) stability of cells or their traces (biomarkers) in a highly radiative habitat, (ii) lithopanspermia hypothesis, contributing to an understanding on the interference between regolith and microorganisms or biomolecules and (iii) planetary protection, to understand if the lunar surface could have been contaminated during past human missions. In addition, the lunar environment can be used as a testbed for the development of bioregenerative life support systems and for long‐term deep‐space exploration missions (Gronstal *et al*., 2007; Crawford, 2010).

In this context, preliminary studies on Earth and in LEO are of the utmost importance for future experiments on the Moon surface and in the Cislunar orbit.

Experiments on the Moon will enable the future human exploration of Mars. It will take a few years before the first experiments are carried out in lunar orbit by the Lunar Gateway platform. Therefore, experiments aboard the ISS are the perfect testbeds for future Moon exploration.

In this study, we investigated the preservation of different classes of biomarkers associated with the extremophilic black fungus *C*. *antarcticus* (isolated from McMurdo Dry Valleys, considered as one of the best terrestrial analogues of Martian surface, Cassaro *et al*., 2021b), grown on LRA after exposure to real space conditions outside the ISS and to preparatory tests of simulated space conditions on the ground. The LRA was previously analysed to compare its mineralogical characteristics with those of Apollo 17 lunar soil from the Maria terrain, as reported by Hill *et al*. (2007). The overall chemical composition of the LRA was as follows: olivine, plagioclase, ilmenite, iron oxide, diopside, hypersthene, apatite and volcanic glass (Table S5; Fig. S1). The chemical compounds of the LRA were similar to those of the lunar soil collected in the Apollo 17 mission, with a difference in concentration (wt.%) (Table S1), except for P_2_O_5_ and Fe_2_O_3_, which were not detected in lunar soil.

First, we focused our attention on melanin pigment detection using confocal Raman and UV–Vis spectrometers. Figure 1 shows the results of confocal Raman microscopy analyses performed on the SVT and SPACE samples. The obtained Raman spectra (Fig. 1A and B respectively) show two typical melanin signals, with a main peak at approximately 1600 cm^−1^ and a second peak near 1340 cm^−1^. According to Pacelli *et al*. (2021), these results show a distinctive fungal melanin peak at approximately 1450 cm^−1^. Fungal melanin pigments are known to be involved in cellular resistance to a multitude of factors, such as toxic metals, hyperosmotic conditions and pH variations (Henson *et al*., 1999), as well as extreme temperatures, desiccation and radiation, including UV light, oxidizing radiation and even ionizing radiation (Eisenman and Casadevall, 2012). Given the universal distribution of melanin in every life domain and its extraordinary resistance, it can be considered a good biomarker for the search for Earth‐like life beyond Earth. Melanic fungi can also be used as radioprotectors in the context of *In Situ* Resource Utilization on the Moon and in future missions to Mars. Active and passive radiation shielding using organic materials has been extensively investigated. For example, extremophilic microorganisms or their macromolecules can be utilized as radioprotectors by leveraging their ability to dissipate radiation. As recently reported by Shunk *et al*. (2021), one strain of the melanized fungus *Cladosporium sphaerospermum* can grow and thrive in the ISS radiation environment, and the strain of this species isolated from the Chernobyl nuclear reactor can use gamma radiation for growth (Dadachova and Casadevall, 2008). In addition, it has been demonstrated that a microbial layer made of this fungus (approximately 1.7 mm) decreases radiation levels by 1.82% and potentially up to ~5% (Shunk *et al*., 2021). This shows the potential role of melanin pigments as components in radiation shields to protect astronauts in future crewed missions to the Moon and Mars, especially considering that during a 3‐year mission to Mars, astronauts would be exposed to 400 mSv of radiation (Letaw *et al*., 1988). Notably, the results obtained for the Top condition (Fig. S2) were different for the SVT and SPACE samples, confirming the need for real space experiments to obtain reliable results in astrobiology.

The stability and detectability of fungal melanin pigments were further confirmed using spectrophotometric analyses. Figure 2A shows the spectral characterization results of melanin pigments with the typical absorption profile of *C*. *antarcticus* melanin pigments (Pacelli *et al*., 2020a). It shows a strong absorption in the UV region (near 230 nm) with a progressive reduction as the wavelength increases, owing to the presence of many complex conjugated structures in the melanin molecule (Cockell and Knowland, 1999). The presence of LRA during the extraction and detection process did not interfere with melanin detection, in contrast to the results obtained by Pacelli *et al*. (2020b), in which the presence of two Martian regolith analogues (P‐MRS: Phyllosilicates Mars Regolith Simulant and S‐MRS: Sulfatic Mars Regolith Simulant) modified the acquired spectra. The implementation of this technique for future instrumentation can be considered for future deep‐space exploration missions. In fact, miniaturized UV–Vis spectrometers have been developed for free‐flying nanosatellites such as the O/OREOS satellite (Bramall *et al*., 2012). Similarly, spectroscopic capability will be used in OREOcube devices, which will measure changes in organic samples when in contact with inorganic surfaces (Sgambati *et al*., 2020).

To verify the resistance of volatile compounds, GC–MS analyses were performed on the SVT and SPACE samples. Overall, the analyses revealed the presence of carboxylic acids, including azelaic, gentisic, lactic and palmitic acids (Table 1), among which only azelaic acid, as previously reported by Pacelli *et al*. (2021), was associated with the presence of *C*. *antarcticus*. Lactic acid was probably derived from the radiolysis of glucose and fructose occurring in the growth medium. As mentioned above, glycerol and ethylene glycol may be derived from extensive degradation of the sugars present in the samples (Deamer and Pashley, 1989).

If life existed or had been transferred in the past to other celestial bodies, such as shadowed craters on the Moon, it may be potentially detectable via nucleic acids, which have, on Earth, an estimated half‐life at −25°C in the order of 10^7^ years (Millar and Lambert, 2013). For example, conditions persisting during the Amazonian period on Mars could have permitted the preservation of DNA beyond 10 million years (Head and Marchant, 2014). The lunar surface is exposed to 570 mSv of galactic cosmic rays in a year, calculated in the period between the solar maximum and minimum (Hayatsu *et al*., 2008), and DNA persistence times under these conditions are not known. Nucleic acids can potentially be preserved for hundreds of thousands to millions of years, especially if they are protected by lunar regolith or present below the surface. In this study, we analysed the stability of nucleic acids after the exposure of fungal colonies mixed with LRA to the radiation rates in LEO during the EXPOSE‐R2 missions.

The effects of exposure on DNA detectability were tested by quantitative nucleic acid amplification based on the principle that DNA lesions can slow down or block the progression of DNA polymerases (Ponti *et al*., 1991). If the amount of starting DNA in a qPCR reaction is the same, DNA with fewer lesions will amplify to a greater extent than the more damaged DNA (Jennerwein *et al*., 1991; Kalinowski *et al*., 1992). For example, DNA from a biological sample exposed to UV radiation will be amplified less than DNA from a corresponding untreated control sample (Van Houten *et al*., 2000). Our results demonstrated a good amplification level for DNA extracted from SVT and SPACE samples (Fig. 3A and B respectively). In addition, we calculated the DNA damage in terms of lesions per kilobase using Poisson distribution. The resulting values (Fig. S4; Table S4, column 6) highlighted a higher lesion frequency in SVT samples than in SPACE samples. These results are in accordance with survival results reported in previous studies. Onofri *et al*. (2019) reported a good survivability of SPACE samples after exposure to real space conditions (86% of survivors for Top exposure), with a decreasing trend as the stress increased. On the other hand, as reported by Pacelli *et al*. (2019), SVT samples recorded a similar trend of survivability after Top, Bottom and Control exposure, but with a lower percentage of survivors (only 22% of vitality measured for Top exposure).

In the context of the obtained results, we could not conclude that nucleic acids could be preserved in a radiation environment similar to that experienced on the lunar surface, but we could conclude that LRA does not interfere with the extraction process and may act as a shield against radiation, mainly if present in the subsurface.

The integration of DNA amplification methods in future space mission payloads could be helpful in detecting traces of DNA‐based life in the subsurface, where the possibility of detecting labile molecules is higher than that on the surface exposed to the radiative environment.

The Search for Extra‐Terrestrial Genomes life‐detection instrument for *in situ* extraction and nanopore sequencing of nucleic acids (Carr *et al*., 2016, 2017) is based on long‐read nanopore sequencing, which is capable of detecting and discriminating Earth nucleic acids and non‐standard nucleic acids (Carr *et al*., 2017), possibly endemic to life on Mars (Ranjan *et al*., 2017). In addition, this type of device has a primary target of searching for extant or recently dead cells encapsulating nucleic acids and can extract nucleic acids from low‐biomass and analogue soils (Mojarro *et al*., 2019). In addition, as reported by Schuerger *et al*. (2019), the short‐target PCR amplification technique could detect traces of dead microbes on the external surfaces of spacecraft that had landed on the Moon, and can hence be used to prevent contamination in the context of planetary protection.

In conclusion, this study is a starting point for future studies beyond LEO (e.g. on the Lunar Gateway) and on the lunar surface. The obtained results shed light on the use of the Moon as a testbed for answering different astrobiological questions, such as the origin of terrestrial life, the limit of life on other celestial bodies and the preservation of different biomolecules in a hostile ionizing environment.

## Experimental procedures

### *Cryomyces antarcticus* and growth medium preparation

The astrobiological test organism used in this experiment was the cryptoendolithic black fungus *C*. *antarcticus* MNA‐CCFEE 515, isolated by R. Ocampo‐Friedmann from sandstone collected at the Linnaeus Terrace (Southern Victoria Land) by H. Vishniac during the Antarctic expedition of 1980–1981, and stored in the Culture Collection of Fungi from Extreme Environments (CCFEE) at the University of Tuscia (Viterbo, Italy), as a section of the Italian National Antarctic Museum.

The substratum for cultivation was prepared as follows: 2% malt extract agar (MEA) was mixed in Petri dishes with 1 g LRA of anorthositic composition mainly dominated by Na‐plagioclase with a density of 1.46 g cm^−4^, calculated using the dry mass volume ratio (Table S5; de Vera *et al*., 2019). The LRA sample was mechanically crushed, and only particles with grain size <1000 μm were selected.

A total of 2000 colony‐forming units of *C*. *antarcticus* were spread and grown on different plates composed of LRA and MEA substratum. Once spread, each plate was incubated in the laboratory for 3 months at 15°C for both experiments. After growth, superficial colonies and the substratum were cut to fit within the wells of the exposure facility in EXPOSE‐R2 (12 mm diameter) and then dehydrated under sterile conditions according to the optimized protocol reported by Pacelli *et al*. (2017).

### Mineralogical composition of LRA

The mineralogical composition of the fraction of the LRA (de Vera *et al*., 2019) used in this study was further characterized by XRF, XRD and SEM‐EDS. The total chemical composition of the major elements (wt.% oxides) was obtained using a Rigaku Supermini X‐ray fluorescence spectrometer, with natural and synthetic standards, and an uncertainty of 0.01% (for sample preparation and analytical details, see Tangari *et al*. (2020)).

XRD measurements of the powdered samples were obtained using a Rigaku MiniFlex II Benchtop XRD System (Rigaku Company, TX, USA) operating at a voltage of 20 kV and a current of 15 mA with CuK_α_ radiation. The mineral phase was identified by comparing the calculated values of interplanar spacing and corresponding intensities of diffraction peaks with theoretical values from the Powder Diffraction File database (JCPDS‐ICDD 2008). The target material of the X‐ray tube was Pd. SEM‐EDS analyses were performed using a JEOL JSM 5400 with an EDS attachment (iXRF Si‐drift detector with ultrathin window) (acc. voltage: 20 keV; carbon‐coated samples).

## Exposure conditions

### Science verification tests

SVTs were performed at the Planetary and Space Simulation Facilities (PSI) at the Institute of Aerospace Medicine (German Aerospace Center, DLR, Koln, Germany). The SVTs were performed before the launch to verify the hardware and the capability of the selected organisms to resist all the stressors experienced during flight exposure within the EXPOSE R2 mission as realistically as possible (de Vera *et al*., 2012; Rabbow *et al*., 2015). SVT exposure was performed using a copy of the flight EXPOSE‐R2 facility. The only differences between the SVT and flight exposure hardware were the missing Monoblock, the interface to the external Russian exposure platform on the ISS, temperature sensors, the manually operated valves, the lack of an R3D‐R2 dosimeter and unpowered trays for the SVT hardware (Rabbow *et al*., 2017). The samples were accommodated in wells with a diameter of 14 mm, within square aluminium carriers having 76 mm sides. Following the accommodation plan scheduled for the EXPOSE‐R2 mission, the SVTs allowed only one replicate per sample and were accommodated in three ground trays. To simulate space‐like conditions, the samples were exposed to a combination of vacuum (10^−5^ Pa), cycling temperatures between −25°C (16 h in the dark) and +10°C (8 h during irradiation), and polychromatic UV (200–400 nm) radiation. The samples were placed in two layers of the exposure carrier. The upper layer sample sets (referred to as ‘Top exposure’) were irradiated with UV radiation, which simulated a mission period of 12 months using the solar simulator SOL2000 at an irradiance of 1271.2 W m^−2^ for an accumulated period of 120 h, applying a total fluency of 5.5 × 10^5^ kJ ms^−2^ (*λ*_200−400_ nm), corresponding to a long‐term space exposure (1 year), previously estimated from EXPOSE data missions and simulations (Rabbow *et al*., 2012, 2015). Below, an identical set of samples was kept in the dark and experienced all simulation parameters except UV radiation (referred to as ‘Bottom exposure’). As in the flight exposure hardware, the trays were closed using MgF_2_ glassware, and tray 1 was filled with nitrogen gas at ambient pressure. Neutral density filters (0.1%) were used to attenuate radiation in all the tests performed. The laboratory and transport control samples were kept in the DLR in the dark at room temperature.

### Exposure in LEO during BIOMEX EXPOSE‐R2 mission

The EXPOSE‐R2 facility was launched from Baikonur to the ISS onboard the Progress cargo spacecraft 56P on 23 July 2014. Outside exposure began on 18 August 2014 and ended on 3 February 2016, after 531 days. The experiment ran for 509 days. For 62 days the samples were kept in the dark for an outgassing step (see Rabbow *et al*., 2017), and for 469 days they were exposed to solar radiation. As for the SVT samples, during the real space conditions in LEO, samples were accommodated in stacks of two sample carriers, where ‘Top exposure’ samples were exposed to solar radiation and ‘Bottom exposure’ samples were shielded from visible and UV radiation. The samples were covered with an MgF_2_ window that screened solar UV radiation with a wavelength of <120 nm. The final total mission UV fluence values were calculated by RedShift Design and Engineering BVBA, Belgium. The preliminary determination of the fluence was 439 and 437 kJ m^−2^, which considers the individual attenuation by the 0.1% additional neutral‐density filters. Both layers were exposed to ionizing radiation derived from solar energetic particles and galactic cosmic rays, and the total mission cosmic ionizing radiation dose experienced was determined using passive detectors; it reached values up to 1 Gy (Berger *et al*., 2017). The LEO vacuum to which samples were exposed ranged from 1.33 × 10^−3^ to 1.33 × 10^−4^ Pa (Rabbow *et al*., 2017) and the temperature fluctuated between 57.98 and −20.9°C. A control set of samples was kept in the laboratory in the dark at room temperature (24°C) and normal ambient pressure.

### Confocal Raman spectroscopy analyses

Raman spectroscopy is a high‐resolution and non‐destructive analytical technique that provides information on the molecular vibration modes of organic or inorganic materials. Confocal Raman analyses were performed directly on the fungal colonies at room temperature and under ambient atmospheric conditions at the German Aerospace Center in Berlin. Before the analyses, the spectrometer (4–5 cm^−1^ spectral resolution) was calibrated using pure silicon and paracetamol test samples. Measurements were taken with a 10× Nikon objective, a 0.25 numerical aperture and a 532 nm excitation laser. Analyses included single spectra to obtain measurements for a single point of the sample, and image scans to investigate a large area of the same sample. Single spectra were recorded at 0.1 mW laser power with 10 s integration and 50 accumulations, whereas image scans were performed at 0.7 mW with 1 s integration and 1 accumulation. Both analyses were performed at three distinct points and areas for a minimum of 1000 measurements per sample.

The limitations of these analyses include potential fluorescence interference, which creates background noise in the spectrum and prevents the detection of Raman peaks. To analyse the Raman spectra, the WITec Project FIVE software was used; spectra were cropped in the region between 200 and 2000 cm^−1^ and then a fifth‐order polynomial function was applied for background subtraction (considered as a combination of many signals contributing to and ‘contaminating’ the spectra recorded). Finally, SNR > 5 masks were applied. For melanin spectra, the SNR is defined as the height of the 1600 cm^−1^ peak divided by the standard deviation, which is considered the spectral region near the main peaks (Pacelli *et al*., 2021).

### Data analysis

### Heat map

An HM is a graphical representation of a set of measurements that allows direct comparison among data through colour maps (Monti *et al*., 2013). In this study, an HM was generated using GraphPad Prism 7 (GraphPad Software, La Jolla, CA, USA, www.graphpad.com), considering the wavelength values of the melanin main peak, measured by Raman spectroscopy for SVT and SPACE samples under all exposure conditions (Top, Bottom and Control), to evaluate any difference in melanin peak positions as a direct consequence of the exposure.

### Fungal melanin extraction and spectrophotometric analysis

To investigate the stability/degradation of fungal pigments, melanin was extracted from the samples using the protocol optimized for *C*. *antarcticus* by Pacelli *et al*. (2020b). The extraction process was followed by the lyophilisation of the pigments. Subsequently, the dried melanin was dissolved in 500 μl of 1 M NaOH; the properties and amounts of extracted melanin were analysed with a VWR‐UV 1600 PC Spectrophotometer using M.Wave professional 2.0 (VWR, Radnor, PA, USA) software. The spectra were recorded in the wavelength range of 200–800 nm using 1 M NaOH as reference. The quantity of extracted melanin was calculated based on a standard curve (at 650 nm) plotted with a concentration of 500 mg ml^−1^ of synthetic DHN melanin (Thermo Fisher Scientific, MA, USA) dissolved in 1 M NaOH (Raman and Ramasamy, 2017).

### Nucleic acids analysis

#### Nucleic acids extraction from LRA and relative amount of DNA lesions

A qPCR assay was performed on extracted DNA to demonstrate the detectability of nucleic acids and to measure their integrity after exposure to simulated space (SVT samples) and real space conditions (SPACE samples).

DNA was extracted from colonies using the Nucleospin Plant kit (Macherey‐Nagel, Düren, Germany) following the protocol optimized for black fungi, as reported by Selbmann *et al*. (2005). Before amplification, DNA was quantified using the QUBIT system and diluted to a concentration of 0.1 ng μl^−1^ for the following analyses. Amplification was performed according to the method described by Pacelli *et al*. (2021). Statistical analyses were performed by one‐way analysis of variance and a pairwise multiple comparison procedure (*t*‐test), carried out using the statistical software SigmaStat 2.0 (Jandel, USA). To calculate the relative amplification rates, the fluorescence readings (of the duplicated samples) were averaged and the blank value (NTC) was subtracted. Damage was expressed mathematically in terms of lesions per kilobase, assuming a Poisson distribution of the lesions. The relative amounts of DNA lesions in 0.9 and 0.33 kb fragments were calculated for samples exposed to SVT and SPACE treatments. Poisson distribution was calculated as follows: lesions/amplicon = −ln(*A*_t_/*A*_o_), where *A*_t_ represents the amplification of treated samples and *A*_o_ is the amplification of untreated controls (Hunter *et al*., 2010).

### Low‐molecular‐weight organic compound detection by GC–MS

To perform GC–MS analyses, each sample was ground using an agate mortar and suspended in 2 ml of ethyl acetate. After 4 h of magnetic stirring at room temperature, the suspension was filtered to remove the solids, and the obtained solution was concentrated under reduced pressure. After extraction and fractionation of the samples with *N*,*N*‐bis‐trimethylsilyl trifluoroacetamide in pyridine (620 μl) at 60°C for 4 h using betulinic acid [3β‐3‐hydroxy‐lup‐20(29)‐en‐28‐oic acid] as the internal standard (0.2 mg), GC–MS was performed. MS was performed as follows: injection temperature, 280°C; detector temperature, 280°C; gradient, 100°C for 2 min and 10°C for 60 min. First, to identify the structure of the products, the spectra were compared with commercially available electron mass spectra libraries such as NIST (Fison, Manchester, UK). The GC–MS analysis was then repeated using standard compounds. All products were recognized with a similarity index greater than 98% compared with that of the reference standards. The analysis was limited to products ≥1 ng ml^−1^, and the yield was calculated as micrograms of the isolated product.

## Author Contributions

S.O., J.P.‐d.V. and E.R. designed the study; A.C., C.P., B.C., M.B. and L.B. performed the experiments. A.C., C.P., B.C., G.G., M.B., U.B., L.B. and R.S. analysed the data. A.C. drafted the paper with inputs from all the authors. All the authors approved the submitted version.

## Supporting information


**Fig. S1.** Representative SEM photomicrograph of the LRA (grain size particles >1000 μm) used in this study, and related representative SEM–EDX spectra of the dominant mineral phases.
**Fig. S2**. Confocal Raman spectroscopy. Signal coverage (%) calculated by applying a SNR mask superior to 5 for A) SVT and B) SPACE samples, for each exposure condition: Top and Bottom, comparing with Control (100%).
**Fig. S3.** Heatmap of the Raman peaks position for A) SVT and B) SPACE samples, for each exposure conditions (Top, Bottom, and Control). Colours scale (on the right) indicate the peak position (cm^−1^) for each image scans.
**Fig. S4.** DNA lesions obtained after Real‐Time PCR amplification of DNA extracted from SVT (on the left) and SPACE samples (on the right). Black bars indicate Top exposure; grey bars indicate Bottom exposure.
**Table S1.** Composition (concentrations expressed in wt. %) of major oxides constituents of the LRA (obtained by X‐ray Fluorescence, XRF) in this study, and the Apollo 17 regolith sample 70,051 (Inductively Coupled Plasma Atomic Emission Spectroscopy, ICP‐AES).
**Table S2.** Concentration (in mg ml^−1^) of extracted melanin from *C*. *antarcticus* colonies exposed to simulated space conditions.
**Table S3.** Mass‐to‐charge ratio (m/z) value and the abundance of peaks of identified compounds
**Table S4.** Representation of the raw Cycle threshold (Ct) values obtained after Real‐time PCR amplification of the LSU and β‐actin genes of DNA extracted from SVT and SPACE samples.
**Table S5.** LRA mineralogical composition (modified from de Vera *et al*., 2019).
