# Supplementary material for: Investigation of fungal biomolecules after Low Earth Orbit exposure: a testbed for the next Moon missions
Source: Environ Microbiol. 2022 Apr 22;24(7):2938–50. doi: 10.1111/1462-2920.15995 (PMC9540993; doi:10.1111/1462-2920.15995)
Supplement: Supplementary file 1 — Fig. S1. Representative SEM photomicrograph of the LRA (grain size particles >1000 μm) used in this study, and related representative SEM–EDX spectra of the dominant mineral phases. Fig. S2. Confocal Raman spectroscopy. Signal coverage (%) calculated by applying a SNR mask superior to 5 for A) SVT and B) SPACE samples, for each exposure condition: Top and Bottom, comparing with Control (100%). Fig. S3. Heatmap of the Raman peaks position for A) SVT and B) SPACE samples, for each exposure conditions (Top, Bottom, and Control). Colours scale (on the right) indicate the peak position (cm−1) for each image scans. Fig. S4. DNA lesions obtained after Real‐Time PCR amplification of DNA extracted from SVT (on the left) and SPACE samples (on the right). Black bars indicate Top exposure; grey bars indicate Bottom exposure. Table S1. Composition (concentrations expressed in wt. %) of major oxides constituents of the LRA (obtained by X‐ray Fluorescence, XRF) in this study, and the Apollo 17 regolith sample 70,051 (Inductively Coupled Plasma Atomic Emission Spectroscopy, ICP‐AES). Table S2. Concentration (in mg ml−1) of extracted melanin from C. antarcticus colonies exposed to simulated space conditions. Table S3. Mass‐to‐charge ratio (m/z) value and the abundance of peaks of identified compounds Table S4. Representation of the raw Cycle threshold (Ct) values obtained after Real‐time PCR amplification of the LSU and β‐actin genes of DNA extracted from SVT and SPACE samples. Table S5. LRA mineralogical composition (modified from de Vera et al., 2019). [file EMI-24-2938-s001.docx]

**The investigation of fungal biomolecules after Low Earth Orbit exposure: a test-bed for the next Moon missions**

Alessia Cassaro^1^, Claudia Pacelli^1,2*^, Mickael Baqué^3^, Barbara Cavalazzi^4,5,6^, Giorgio Gasparotto^4^, Raffaele Saladino^1^, Lorenzo Botta^1^, Ute Böttger^7^, Elke Rabbow^8^, Jean-Pierre de Vera^9^ and Silvano Onofri^1^

^1^Department of Ecological and Biological Sciences, University of Tuscia, Largo dell’Università snc, 01100 Viterbo, Italy

^2^ Human spaceflight and Scientific Research Unit, Italian Space Agency, via del Politecnico, 00133 Rome, Italy

^3^German Aerospace Center (DLR), Institute of Planetary Research, Planetary Laboratories department, Rutherfordstraße 2, Berlin, Germany

^4^Department of Biological, Geological and Environmental Sciences, University of Bologna, Via Zamboni 67, 40126 Bologna, Italy

^5^Department of Geology, University of Johannesburg, Auckland Park, 2006 Johannesburg, South Africa

^6^Le Studium Loire Valley Institute for Advanced Studies, Rue Dupanloup 1, Orléans, France

^7^German Aerospace Center (DLR), Institute of Optical Sensor Systems, Rutherfordstraße 2, Berlin, Germany

^8^German Aerospace Center (DLR), Institute of Aerospace Medicine, Radiation Biology, Linder Höhe, 51147 Cologne, Germany

^9^Space Operations and Astronaut Training, MUSC, German Aerospace Center (DLR), Linder Höhe, 51147 Cologne, Germany

**Supplementary information**

**Results**

- **Mineralogical composition of LRA**
- **Detection of possible alterations in melanin pigments by Confocal Raman spectroscopy and Heat map analyses**
- **Detection of melanin pigments by spectrophotometric analyses**
- **Low-molecular-weight organic compounds detection by Gas Chromatography-Mass Spectrometry**
- **Investigation of nucleic acids by qPCR assay and relative amount of DNA lesions**

**Material and Methods**

- **Mineralogical composition of LRA**

**Mineralogical composition of LRA**

**Figure S1.** Representative SEM photomicrograph of the LRA (grain size particles >1000 μm) used in this study, and related representative SEM-EDX spectra of the dominant mineral phases.


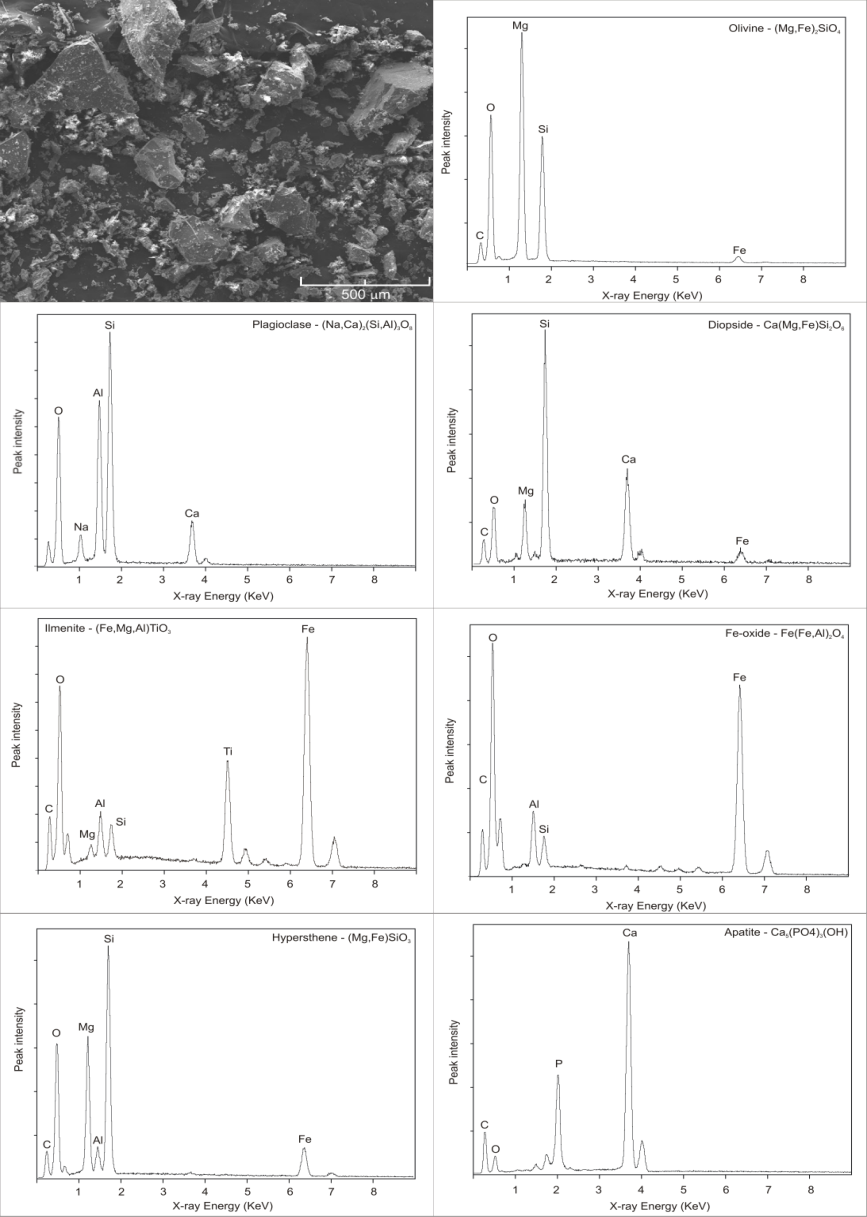


**Table S1.** Composition (concentrations expressed in wt. %) of major oxides constituents of the LRA (obtained by X-ray Fluorescence, XRF) in this study, and the Apollo 17 regolith sample 70051 (Inductively Coupled Plasma Atomic Emission Spectroscopy, ICP-AES).

| **Constituent oxides (wt. %)** | **Concentration**  **(wt. %)** | | |
| --- | --- | --- | --- |
|  | **Lunar regolith analog**  **(XRF)** | **Apollo 17 70051 regolith sample**  **(ICP-AES)*** | |
| SiO_2_ | 51.78 | | 42.2 |
| TiO_2_ | 0.61 | | 5.1 |
| Al_2_O_3_ | 23.29 | | 15.17 |
| Fe_2_O_3_ | 3.54 | | 13.8° |
| MnO | 0.06 | | 0.15 |
| MgO | 3.70 | | 10.3 |
| CaO | 9.63 | | 11.5 |
| Na_2_O | 5.46 | | 0.24 |
| K_2_O | 1.01 | | 0.07 |
| P_2_O_5_ | 0.65 | | --- |
|  |  | |  |
| Ni | 221 (ppm) | | NA 140 (<45; Table 2) |
| Zn | 62 (ppm) | | NA 53 |
| Sr | 906 (ppm) | | NA 167 |
| Y | 26 (ppm) | | NA 54 |

**Hill et al., 2007*

*°Fe as FeO (12.4); recalculated as Fe_2_O_3_*

**Detection of possible alterations in melanin pigments by Confocal Raman spectroscopy and Heat map analyses**

**Figure S2. Confocal Raman spectroscopy.** Signal coverage (%) calculated by applying a SNR mask superior to 5 for **A)** SVT and **B)** SPACE samples, for each exposure condition: Top and Bottom, comparing with Control (100%).


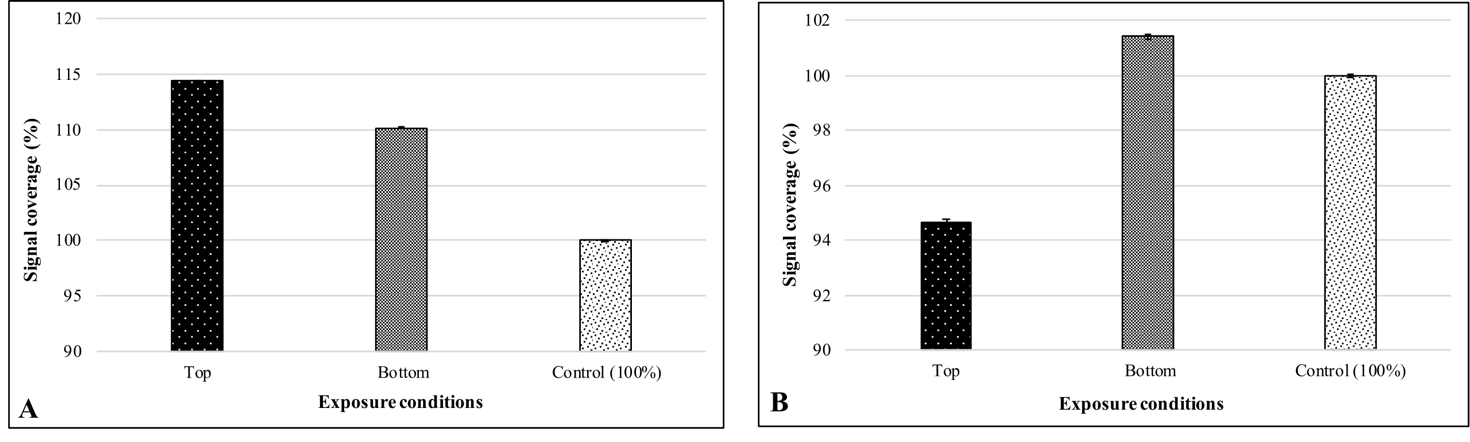


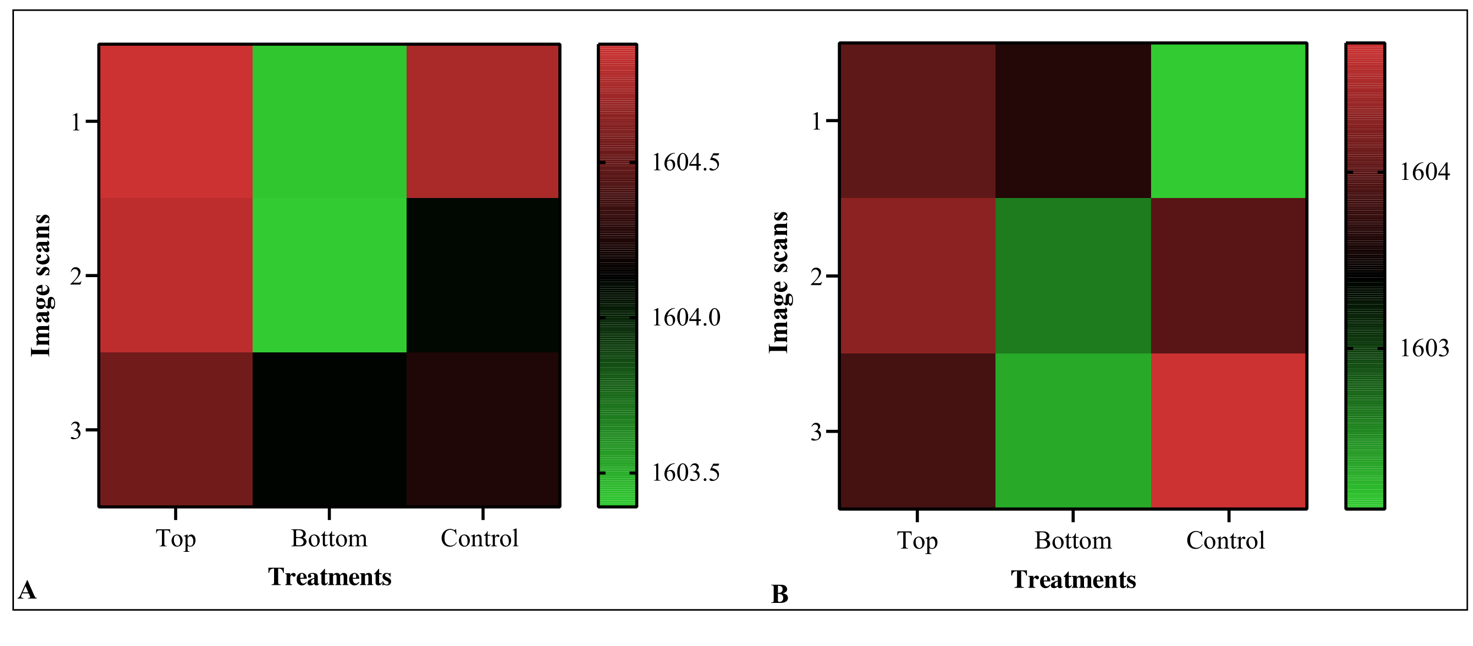
**Figure S3.** Heatmap of the Raman peaks position for **A)** SVT and **B)** SPACE samples, for each exposure conditions (Top, Bottom, and Control). Colors scale (on the right) indicate the peak position (cm^-1^) for each image scans.

**Detection of melanin pigments by spectrophotometric analyses**

**Table S2.** Concentration (in mg/ml) of extracted melanin from *C. antarcticus* colonies

exposed to simulated space conditions.

| **Sample**  **exposure** | **Concentration**  **(mg/ml)** |
| --- | --- |
| Top | 0.45095 |
| Bottom | 0.943527 |
| Control | 1.90093 |

**Low-molecular-weight organic compounds detection by Gas Chromatography-Mass Spectrometry**

**Table S3.** Mass-to-charge ratio (m/z) value and the abundance of peaks of identified compounds

| **Products^[a]^** | **m/z (%)** |
| --- | --- |
| **Azelaic Acid^[c]^** | 317 (25) [M-CH_3_], 302 (3) [M-2xCH_3_], 243 (2) [M-OSi(CH_3_)_3_], 201 (15) [M-Si(CH_3_)_3_-CO_2_-CH_3_], 186 (3) [M-2xSi(CH_3_)_3_], 170 (4) [M-OSi(CH_3_)_3_- Si(CH_3_)_3_], 73 (100). |
| **Gentisic acid^[d]^** | 370 (5) [M], 355 (100) [M-CH_3_], 267 (10) [M- Si(CH_3_)_3_-2xCH_3_], 223 (10) [M-2xSi(CH_3_)_3_]. |
| **Palmitic Acid^[b]^** | 328 (20) [M], 313 (100) [M-CH_3_], 73 (100). |
| **Lactic Acid^[b]^** | 219 (6) [M-CH_3_], 190 (14) [M-CO_2_], 147 (71) [M-Si(CH_3_)_3_-CH_3_], 133 (7), 117 (76) [M-Si(CH_3_)_3_-(CH_3_)_3_]. |
| **Glucose^[e]^** | 437 (5) [M-Si(CH_3_)_3_-2xCH_3_], 394 (4) [M-2xSi(CH_3_)_3_], 305 (5) [M-OSi(CH_3_)_3_-2xSi(CH_3_)_3_], 217^g^ (30), 204^g^ (100), 191^g^ (75). |
| **Fructose^[e]^** | 437 (5) [M-Si(CH_3_)_3_-2xCH_3_], 217^g^ (30), 204^g^ (100), 146 (75). |
| **Glucitol^[f]^** | 319 (60) [M], 297 (94) [M-CH_3_], 282 (30) [M-2xCH_3_], 267 (40) [M-3xCH_3_]; 217^g^ (90), 204^g^ (80); 147 (40). |
| **Glycerol^[d]^** | 293 (3) [M-CH_3_], 263 (2) [M-3xCH_3_], 218 (20) [M-OSi(CH_3_)_3_], 205 (60) [M-OSi(CH3)_3_-CH_3_], 191 (3) [M-OSi(CH_3_)_3_-2xCH_3_], 171 (4) [M-OSi(CH_3_)_3_-3xCH_3_]. |
| **Ethylene Glycol^[c]^** | 191 (25) [M-CH_3_], 147 (100) [M-4xCH_3_], 133 (5) [M-Si(CH_3_)_3_], 103 (20) [M-Si(CH_3_)_3_-2xCH_3_]. |

**[a]** Mass spectroscopy was performed by using a GC-MS Varian 410 GC-320 MS. The peak abundance is reported in parenthesis **[b]** Product analysed as the monosilyl derivative; **[c]** Product analysed as the bis-silyl derivative; **[d]** Product analysed as the tris-silyl derivative; **[e]** Product analysed as the penta-silyl derivative; **[f]** Product analyzed as the hexa-silyl derivative; **[g]** Ions characteristic for EI/MS sugar degradation: *m/z* 217 [(CH_3_)_3_SiOCH=CH-CH=OSi(CH_3_)_3_]^+^, *m/z* 204 [(CH_3_)_3_SiOCH=CHOSi(CH_3_)_3_]^+•^, *m/z* 191 [(CH_3_)_3_SiOCH=OSi(CH_3_)_3_]^+^.

**Investigation of nucleic acids by qPCR assay and relative amount of DNA lesions**

**Figure S4.** DNA lesions obtained after Real-Time PCR amplification of DNA extracted from SVT (on the left) and SPACE samples (on the right). Black bars indicate Top exposure; grey bars indicate Bottom exposure.

**
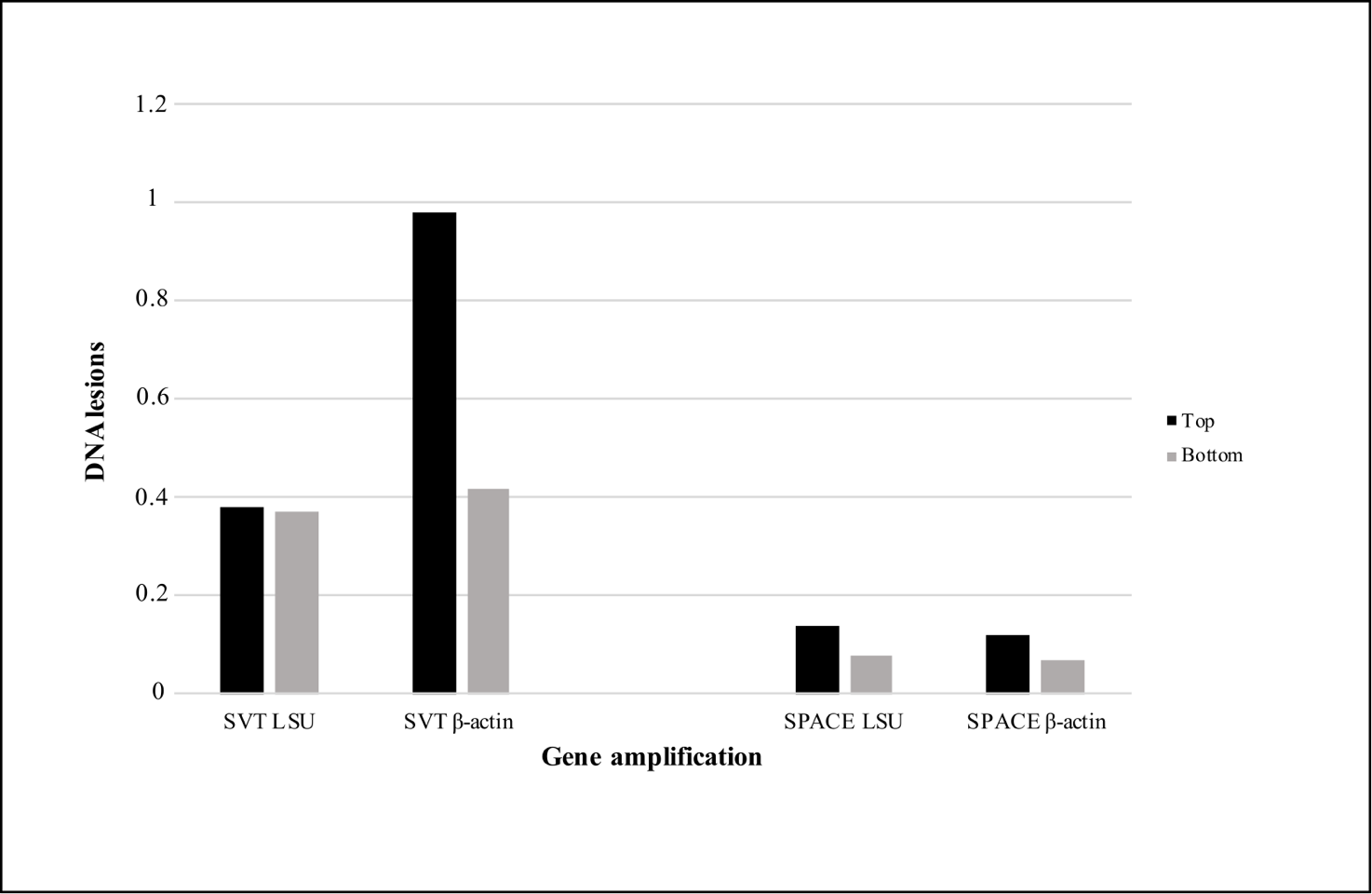
**

**Table S4.** Representation of the raw Cycle threshold (Ct) values obtained after Real-time PCR amplification of the LSU and β-actin genes of DNA extracted from SVT and SPACE samples.

| **SVT LSU** | | | | | |
| --- | --- | --- | --- | --- | --- |
| **Samples** | **Mean** | **Final read** | **Relative**  **amplification** | **Lesion frequency** | **Lesion/0.9 kb** |
| **Top** | 26.21 | -9.72 | 0.65 | 0.42 | 0.38 |
| **Bottom** | 26.08 | -9.85 | 0.66 | 0.41 | 0.37 |
| **Control** | 21.08 | -14.85 | 1 | 0 |  |
| **NTC** | 35.93 |  |  |  |  |
| **SVT β-actin** | | | | | |
| **Samples** | **Mean** | **Final read** | **Relative**  **amplification** | **Lesion frequency** | **Lesion/0.33 kb** |
| **Top** | 29.51 | -0.33 | 0.05 | 2.97 | 0.98 |
| **Bottom** | 28.04 | -1.80 | 0.29 | 1.27 | 0.42 |
| **Control** | 23.44 | -6.40 | 1 | 0 |  |
| **NTC** | 29.84 |  |  |  |  |

| **SPACE LSU** | | | | | |
| --- | --- | --- | --- | --- | --- |
| **Samples** | **Mean** | **Final read** | **Relative amplification** | **Lesion frequency** | **Lesion/0.9 kb** |
| **Top** | 26.11 | -11.93 | 0.86 | 0.15 | 0.14 |
| **Bottom** | 25.29 | -12.75 | 0.92 | 0.08 | 0.08 |
| **Control** | 24.15 | -13.89 | 1 | 0 |  |
| **NTC** | 38.04 |  |  |  |  |
|  |  |  |  |  |  |
| **SPACE β-actin** | | | | | |
| **Samples** | **Mean** | **Final read** | **Relative amplification** | **Lesion frequency** | **Lesion/0.33 kb** |
| **Top** | 24.84 | -3.41 | 0.71 | 0.34 | 0.12 |
| **Bottom** | 24.27 | -3.98 | 0.83 | 0.19 | 0.06 |
| **Control** | 23.46 | -4.80 | 1 | 0 |  |
| **NTC** | 28.25 |  |  |  |  |

Column one, sample identification; column two, average of Ct values from qPCR amplification; these values are corrected with background (no template control: NTC) subtraction (column 3). Relative amplification (column 4) is calculated comparing the values of the treated samples with untreated control. Lesion frequency (column 5) is obtained based on the values plotted on column 4 and are expressed as lesions per 0.9 and 0.33 kb of DNA amplification (column 6 and Fig. S3).

**Material and Methods**

**Mineralogical composition of LRA**

**Table S5.** LRA mineralogical composition (modified from de Vera et al., 2019).

| **Mineral phases** | **Weight (%)** |
| --- | --- |
| olivine (Fo_96_) | 5.7 |
| diopside | 8.9 |
| hypersthene | 5.7 |
| plagioclase | 66.8 |
| apatite | 1.1 |
| ilmenite | 1.1 |
| iron | 1.3 |
| volcanic glass | 9.4 |
| **Density: 1.46 g/cm^-3^** | |
